# Source Attribution of PM_2.5_ Health Benefits Over Northern Hemisphere Using Adjoint of Hemispheric CMAQ

**DOI:** 10.1029/2025GH001533

**Published:** 2026-01-20

**Authors:** Y. B. Oztaner, S. Zhao, B. Henderson, R. Mathur, A. Hakami

**Affiliations:** ^1^ Department of Civil and Environmental Engineering Carleton University Ottawa ON Canada; ^2^ U.S. Environmental Protection Agency Research Triangle Park NC USA

**Keywords:** adjoint sensitivity analysis, PM_2.5_ health impacts, northern hemisphere

## Abstract

The adjoint of the U.S. EPA's Community Multiscale Air Quality (CMAQ) model is extended for hemispheric scale applications and is used to estimate location‐specific health impacts from primary PM_2.5,_ and PM_2.5_ precursor emissions (NH_3_, NO_X_ and SO_2_). We estimate the monetized health burden due to mortality caused by chronic PM_2.5_ exposure among adults living in the northern hemisphere, using a generalized concentration‐response function. The health impact sensitivities show large spatial variability over the northern hemisphere and exhibit a great deal of seasonal variability, especially for inorganic precursor emissions. The largest marginal impacts are seen for NH_3_ and primary PM_2.5_. The estimated health impacts for a 10% reduction in emissions reveal a hemispheric burden of 513,700 avoided mortality and monetized health benefits at above 1.2 trillion USD_2016_. The largest regional contribution to hemispheric mortality is found to be in East and South Asia, particularly China and India (183,760 and 123,440 for a 10% reduction in emissions, respectively). Monetized health burdens are estimated to be highest in China and Europe (∼365 and ∼252 million USD for a 10% reduction in emissions) while it is relatively similar in India (∼175 million USD) as in Canada and the United States (∼177 million USD). Sectoral source contribution analysis demonstrates that the agriculture (19%) and residential (15%) sectors are the largest contributors to the northern hemispheric scale health burden, however, regional differences exist in the results. Examining location‐ and sector‐specific health impacts can inform more effective regulatory measures.

## Plain Language Summary

Chronic exposure to outdoor PM_2.5_ is one of the highest mortality risk factors. We used an air quality model to understand how reducing air pollution could improve health across the Northern Hemisphere. We estimated how many deaths could be avoided and the economic value of these health benefits if emissions were reduced. We looked at primary PM_2.5_ emissions and gases like ammonia, nitrogen oxides, and sulfur dioxide, which can form PM_2.5_ in the air. Using a sophisticated sensitivity analysis, we linked emissions to health outcomes and economic impacts in specific locations. Our results show that health impacts vary by season and region, and that ammonia and primary PM_2.5_ emissions cause the greatest harm. Reducing emissions by 10% could prevent more than 500,000 premature deaths each year and save over $1.2 trillion (USD 2016). East and South Asia had the highest number of deaths, while China and Europe faced the greatest economic burden. Emissions from agriculture and residential sources were the biggest contributors to health impacts. Identifying where pollution reductions would bring the most health benefits can support the development of more targeted and effective air quality policies.

## Key Points

- We extend the multiphase adjoint of CMAQ to the hemispheric scale to assess health impacts from chronic exposure to PM_2.5_
- Monetized health benefits and mortality estimates are attributed to emission source locations over northern hemisphere
- The largest contribution to health burden over the northern hemisphere is found to be in East and South Asia, particularly China and India

## 1 Introduction

The global death toll estimates due to ambient air pollution range between 3 and 10 million (varies between 7% and 18% of global mortality; Burnett et al., 2018; Cohen et al., 2017; HEI, 2020; Lelieveld et al., 2015, 2019; Murray et al., 2020; Sang et al., 2022; Vohra et al., 2021; WHO, 2021). Chronic exposure to outdoor fine particulate matter with an aerodynamic diameter equal to or less than 2.5 μm (PM_2.5_) remains the main contributor, with the GBD 2021 study estimating nearly 90% of global mortality attributed to ambient air pollution (Brauer et al., 2024), while a recent work suggests that nitrogen dioxide (NO_2_) may also contribute substantially to premature mortality (Camilleri et al., 2023). Additionally, the mortality estimates due to exposure to ambient PM_2.5_ concentrations are higher in newer epidemiological studies (Burnett et al., 2018; Heft‐Neal et al., 2018). The latest Global Burden of Disease Study (GBD) 2021, estimates 4.71 million mortality attributable to ambient PM_2.5_ exposure in 2021, which is a 94% increase compared to the estimate for 1990 (Brauer et al., 2024).

Ambient PM_2.5_ concentrations and PM_2.5_‐related health impacts are often treated as a local/regional problem (Fann et al., 2009, 2011, 2012). However, the contribution of intercontinental transport of air pollution to ambient PM_2.5_ pollution is not negligible. Anenberg et al. (2014) estimate 3%–7% of PM_2.5_‐related mortality occurs outside of source regions linked to PM_2.5_ concentrations transported between continents, while more recent studies report even larger contributions, up to 12% (Zhang et al., 2017) and 14% (Chen et al., 2022). Intercontinental contribution to regional PM pollution creates an extra health burden and suggests that mortality associated with PM pollution is not a regional problem alone (Im et al., 2018; Liu et al., 2009, 2020; Zhang et al., 2017). Environmental policies to achieve lower PM_2.5_ concentrations carry a significant price tag regardless of the scale (i.e., cities, provinces, countries, etc.). Atmospheric chemical transport models (CTMs) are tools of choice to assess how ambient concentrations are affected by changes in emissions prior to promulgating regulations and implementing policies. In that capacity, CTMs are often used for estimating various measures of health impacts (e.g., mortality counts, societal costs, or benefits per ton (BPTs)) of reducing anthropogenic emissions by incorporating epidemiological and/or economic models and data. The most traditional approach to estimate the impact of emissions is brute‐force (BF) sensitivity analysis (Anenberg et al., 2014; Im et al., 2018). Although the BF approach provides valuable perspectives, it can be prone to numerical inaccuracies (Napelenok et al., 2006) and is computationally expensive to execute if the response to a large number of emission change options is required. Decouple Direct Method (DDM), a formal forward sensitivity method (Dunker, 1981; Hakami et al., 2003; Tagaris et al., 2010; Yang et al., 1997) offers higher computational efficiency than BF but ultimately carries the same limitation in face of large number of sources or control options.

Reverse influence modeling, or adjoint sensitivity analysis, is a complementary approach, that is, particularly powerful and efficient in estimating impacts from a large number of sources. Adjoint models trace back influences (sensitivities) from a predefined receptor area to each source location at preceding timesteps, that is, backward in time. While adjoint versions have been developed for a number of CTMs (Elbern & Schmidt, 1999; Guerrette & Henze, 2015; Hakami et al., 2005, 2007; Henze et al., 2007; Mallet & Sportisse, 2005; Martien & Harley, 2006; Menut, 2003; Menut et al., 2000; Sandu et al., 2005; Zhao et al., 2020), most health‐based applications of the adjoint method have been limited to GEOS‐Chem and CMAQ models (Koo et al., 2013; Lee et al., 2015, 2017; Nawaz et al., 2021; Oztaner et al., 2024; Pappin et al., 2015; Pappin & Hakami, 2013a, 2013b; Qu et al., 2022).

Here, we present the extension of the multiphase (gas‐ and particulate phase) adjoint of CMAQ (CMAQ‐ADJ) to the hemispheric scale, accompanied by enhancements for better representation of long‐range transport. We apply the adjoint of Hemispheric CMAQ (H‐CMAQ) to estimate source contributions to the hemispheric health burden of chronic PM_2.5_ exposure. We estimate location‐specific source impacts, in the form of mortality sensitivities and their monetization (i.e., BPTs) over the northern hemisphere. We also assess the sectoral and seasonal contributions of the estimated source impacts from primary PM_2.5_ and inorganic precursor emissions.

## 2 Methods

### 2.1 Modeling Setup

Long‐term PM_2.5_‐related health impacts are estimated for emissions of primary PM_2.5_ (elemental carbon, organic carbon, sulphate, ammonium, fine particulate) and PM_2.5_ precursor emissions (SO_2_, NO_X_ and NH_3_) using CMAQ v5.0 and its multiphase adjoint. The current version of CMAQ Adjoint does not yet incorporate the more recent oxidation pathways involved in secondary organic aerosol (SOA) formation, and therefore underestimates SOA component. Similarly, this version treats primary organic emissions as non‐volatile, and as such some of the SOA burden is captured as primary PM_2.5_ burden. CMAQ is applied on a modeling domain covering the northern hemisphere using a polar stereographic projection with horizontal grid spacing of 108 km (187 × 187 grids) and a 44‐layer configuration vertically. Applying a 44‐layer configuration on the hemispheric scale better portrays long‐range transport in the free troposphere and stratospheric‐tropospheric exchange processes (Mathur et al., 2017). The U.S. EPA's 2016 Hemispheric Emission modeling platform emissions are used in the study which includes anthropogenic, biomass burning, biogenic dust and sea salt sources. More details about the emission inventory can be found elsewhere (US EPA, 2019). The CMAQ and its adjoint model are driven by meteorological inputs from the Weather Research and Forecasting model (WRFv3.8.1; Skamarock et al., 2008) for the year 2016. The year 2016 was selected because the U.S. EPA has tested and evaluated the 2016 emission platform extensively (e.g., Mathur et al., 2017; Skipper et al., 2024). Adjoint simulations on a hemispheric scale require longer simulation periods (i.e., a year) to account for global circulation patterns. Therefore, the adjoint simulations in this study are conducted for the year 2016 to capture a year‐long impact of atmospheric circulation on the emission influences over the northern hemisphere. CMAQ simulations are conducted at an hourly temporal resolution. The adjoint sensitivities for each emitted species are therefore computed at the same hourly scale. The simulation period includes 6 months of forward simulation spin‐up and 2 weeks of backward spin‐up time. The model performance analysis shows an overall mean bias of −8.3 μg/m^3^, and the results are provided in SI (Table S1 and Figure S9 in Supporting Information S1).

### 2.2 Enhancement to Organic Nitrate Chemistry of the Adjoint

The 2005 carbon bond chemical mechanism with updated toluene chemistry (CB05TU) is used for initial hemispheric scale CMAQ simulations. In the CB05TU mechanism, the organic nitrate species are presented by the species NTR. NTR plays a critical role in reallocating NO_X_ from source areas to remote areas. Recycling of NO_X_ through reservoir organic nitrate species can help to better present the chemistry of NO_X_ reservoirs; however, expanded NTR chemistry is not available in the original CMAQ 5.0 adjoint (Zhao et al., 2020). To better characterize NOx source and sink locations in the hemispheric‐scale CMAQ application, the gas‐phase chemistry is updated to include the expanded NTR chemistry. The single NTR species is replaced with seven NTR species. The dry deposition velocity and wet scavenging for NTR species are mapped to HNO_3_, as suggested by Mathur et al. (2017). The chemical kinetic mechanism implemented in the CMAQ‐ADJ is updated to CB05TU from CB05 for this study using The Kinetic PreProcessor v2.1 (KPP; Damian et al., 2002). The updated mechanism in adjoint model is evaluated against the BF Finite Difference Method (FDM) for gas‐phase chemistry. The results of adjoint model evaluation for gas‐phase chemistry are given in Figures S1 and S2 of Supporting Information S1.

### 2.3 Source Attribution of PM_2.5_ Health Benefits

We apply adjoint sensitivity analysis to attribute long‐term PM_2.5_ health impact over the northern hemisphere. Adjoint source attribution requires a scalar, policy‐based metric as the adjoint cost or objective function. The adjoint system of equations is then driven by the derivatives of the cost function, called adjoint forcing, to trace the influence (sensitivities) back in time and space to the source locations, providing location‐specific sensitivities to a change in emissions. Here, we define two cost functions: mortality and its monetization. We employ the Global Exposure Mortality Model (GEMM; Burnett et al., 2018) which provides a generalized concentration response function (CRF) for chronic exposure mortality from non‐communicable diseases (NCDs) and lower respiratory infections (LRIs) based on several cohort analyses across the globe (Equations (1), (2), (3), (4)). The GEMM tends to produce higher estimates for PM_2.5_ mortality; however, a previous study for North America finds that source impact estimates based on GEMM are generally comparable to those that are based on other North American epidemiological models (Hakami, Zhao, Soltanzadeh, et al., 2024; Zhao et al., 2024).

(1) $$J=\sum_{i}M_{0,i}\times P_{i}\left(1-e^{-\theta T\left(z\right)}\right),$$

where,

(2) $$Τ\left(z\right)=\log\left(1+\frac{z}{\alpha }\right)\omega \left(z\right),$$

(3) $$\omega \left(z\right)=\frac{1}{1+e^{-\left(z-\mu \right)/\nu }},$$

and

(4) $$z=\mathrm{MAX}\left(0,{\mathrm{PM}}_{2.5}-C_{f}\right)$$

with the coefficients: $\theta =0.1231,\alpha =1.5,\mu =10.4,\nu =25.9\;\text{and}\;C_{f}=2.4\;\mu g/m^{3}$.

J is mortality as the first cost function while $M_{0}$ is the baseline mortality rate (BMR) and P is the population for each location *i*. We calculate the adjoint forcing, the derivative of J with respect to model concentrations. To be consistent with GEMM, country‐specific health endpoints' (NCD + LRI) baseline mortality rates (BMR, M_0_) for adults 25 years and older are obtained from IHME (Institute for Health Metrics and Evaluation, 2021) for the simulation year. The gridded population data (25+ age) for the adjoint forcing is retrieved from the WorldPop database (WorldPop, 2021) at a finer spatial resolution (1 km), and aggregated to the model's horizontal grid spacing of 108 km.

Monetization of mortality counts is based on the value of statistical life (VSL). VSL is based on a willingness‐to‐pay estimate for a small reduction in mortality risk. While there is lack of consistent empirical studies assessing VSL across the world, country‐specific or region specific VSLs have been used previously in cost‐benefit analyses of the previous studies (Markandya et al., 2018; Shindell et al., 2012; West et al., 2013). We apply the unit value transfer approach as recommended by the Organization for Economic Cooperation and Development (OECD, 2012, 2014). This approach adjusts the recognized VSL of the OECD for 2005 to all countries based on their gross domestic products (GDPs) per capita, income growth rates, consumer price indices and income elasticity. Country‐specific VSL is calculated with the formula recommended by the OECD (OECD, 2012, 2014):

(5) $${\text{VSL}}_{c,2016}={\text{VSL}}_{\text{OECD},2005}\times {\left(\frac{Y_{c,2005}}{Y_{\text{OECD},2005}}\right)}^{b}\times {\left(1+\%\Delta P+\%\Delta Y\right)}^{b}$$

Where ${\text{VSL}}_{c,2016}$ is the VSL for country *c*, ${\text{VSL}}_{\text{OECD},2005}$ is the base value; $Y_{\text{OECD},2005}$ is the average GDP per capita of the OECD, $Y_{c,2005}$ is the GDP per capita for country *c*, %ΔP is the consumer price index (CPI) and %ΔY is the income growth rate, retrieved from the World Bank Database (The World Bank, 2021). An income elasticity (b) of 0.8 is used as recommended by OECD (OECD, 2014).

Adjoint results for monetized mortality cost function are estimates of BPTs and number of avoided mortality sensitivities for each emitted species at every hour. Model outputs for two adjoint cost functions are postprocessed at the model's horizontal grid spacing 108 km for every hour and are aggregated temporally to estimate annual source impacts of primary PM_2.5_ and PM_2.5_ precursor emissions. Note that PM_2.5_ species are aggregated based on emission weighting to estimate primary PM_2.5_ annual source impacts. It should be noted that calculation of BPTs and mortality sensitivities are subject to multiple sources of uncertainty including modeling inputs, model formulation and study design. Using a similar methodology as this work over a North American domain, Hakami, Zhao, Vasilakos, et al. (2024) examined uncertainties that stem from various choices and assumptions in adjoint‐based source impact estimations. They found that grid resolution may significantly affect source impact estimates, while the choice of CRF can introduce uncertainties that are as large as the statistical uncertainty (i.e., confidence interval) for each CRF (Hakami, Zhao, Soltanzadeh, et al., 2024; Hakami, Zhao, Vasilakos, et al., 2024). They also find that episodic representation of seasonal source impacts adds only low‐to‐moderate levels of uncertainty.

## 3 Results

### 3.1 Hemispheric and Regional Burden

**Figure 1:**
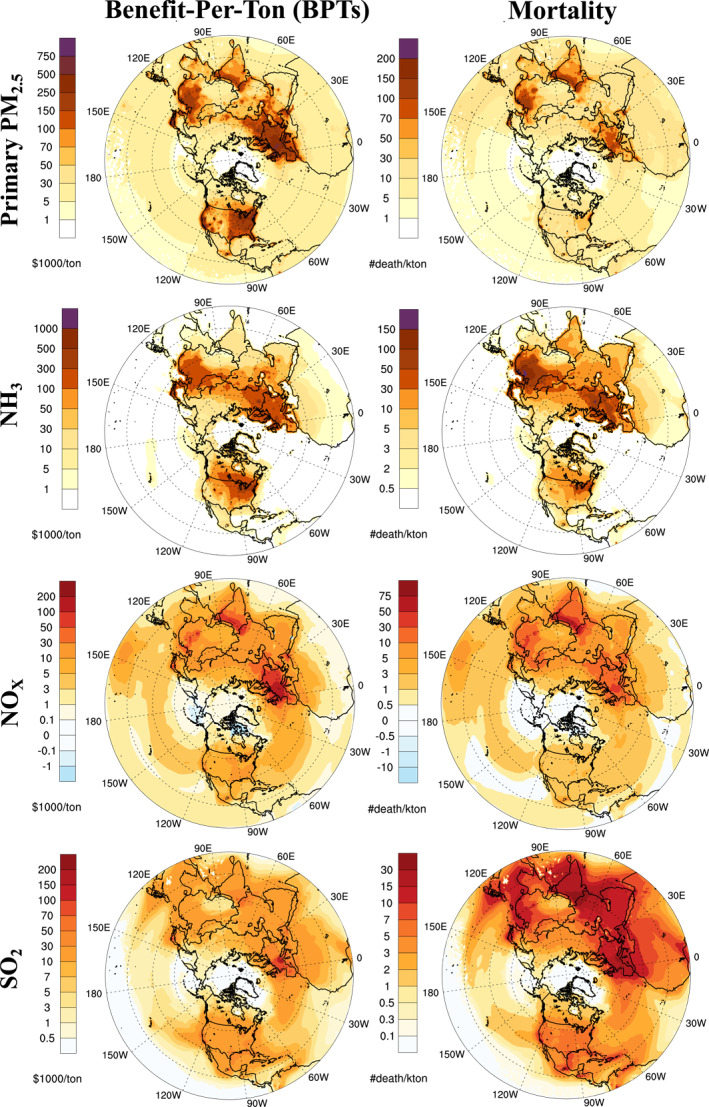
Estimated annual BPTs (USD_2016_) for a reduction in primary PM_2.5_ and precursor PM_2.5_ emissions (NH_3_, NO_X_ and SO_2_) emitted from surface sources (left panel) and estimated annual avoided mortality counts (number of deaths) attributable to a kton reduction in primary PM_2.5_ and its precursor emissions in each location (right panel).

Figure 1 shows mortality sensitivities (death/kiloton, or #/kton) and their monetization, that is estimated annual BPTs ($USD/ton), for surface emissions across the northern hemisphere. Mortality sensitivities and BPTs from elevated emissions are given in Figure S3 of Supporting Information S1. BPTs range between −$28,000 for a ton reduction of NO_X_ to $1,400,000 for a ton reduction in NH_3_ emissions. The highest BPT is found to be for NH_3_ near Moscow; however, BPTs for primary PM_2.5_ emissions show overall larger values. Similarly, the highest sensitivities of mortality are estimated for NH_3_ near Moscow and east China. Mortality sensitivities range from −1 avoided death for a kiloton reduction of NO_X_ to 380 avoided deaths for a kiloton reduction of NH_3_ emissions.

Annual BPTs and mortality sensitivities have positive responses for primary and inorganic precursor emissions, except for NO_X_ emissions. The negative BPTs and mortality from NO_X_ are estimated in some regions (i.e., near Shanghai, Hong Kong) where the NO_X_‐inhibited regime leads to small but negative source impacts. These negative sensitivities are mainly due to NO_X_ titration of ozone, coupled with the subsequent role of ozone in night‐time inorganic nitrate formation. Overall, BPT and mortality estimates show a high level of spatial variability for all species, indicating the value of location‐specific source impact estimates. The spatial distribution of sensitivities for primary PM_2.5_ follows the population distribution, unlike sensitivities from precursor PM_2.5_ emissions. Note that the results shown in Figure 1 are source impacts from unit emissions at any location, whether or not that location has actual emissions. In other words, source impact estimates (e.g., BPTs) can be non‐zero or even large in places where no sources exist. Locations over the ocean show positive BPT estimates due to subsequent atmospheric transport to more heavily populated areas. Mortality sensitivities from primary PM_2.5_ emissions are high in China and northern India; however, Europe and North America have comparably large BPT estimates to India and China, due to their larger VSLs.

Our results also reveal that seasonal variability in BPT and mortality sensitivities are significant, particularly for inorganic precursor emissions (see Figures S4–S7 in Supporting Information S1). Seasonal variability for inorganic precursors is primarily due to the dependence of formation processes and partitioning on temperature and other weather parameters. Of particular note, ammonia exhibits significantly larger sensitivities in winter, where its emissions are seasonally lowest. Mortality sensitivities reach up to 650 deaths per kton of NH_3_ emissions in winter, compared to a maximum of 155 deaths/kton in summer. Primary emissions of PM_2.5_ show significantly lower variability across seasons, as they are emitted directly into the atmosphere.

Figure 1 shows marginal source impacts on mortality or its monetary valuation. However, the overall burden of emissions will depend on both location‐specific marginal source impacts (e.g., BPTs), and emission rates at various locations. Table 1 shows the monetized health outcomes (health benefits) and avoided mortality for a 10% reduction in primary PM_2.5_ and inorganic precursor emissions by regions for surface and elevated source emissions (anthropogenic and natural sources). The 10% emission reduction level is chosen as a range over which the marginal estimates are less prone to the presence of expected nonlinearities.

**Table 1:** Regional Aggregated Monetized Health Benefits and Total Avoided Mortality for a 10% Reduction in Primary PM_2.5_ and PM_2.5_ Precursor Emissions (All Natural and Anthropogenic Sources)

Region/Pollutant	Surface				Elevated				Total
	PM_2.5_	NH_3_	NO_X_	SO_2_	PM_2.5_	NH_3_	NO_X_	SO_2_	
India **USD**_**2016**_ **‐million** (avoided death)	**49,550** (37,390)	**14,490** (11,210)	**27,390** (20,530)	**3,380** (2,820)	**26,840** (16,540)	**660** (390)	**15,330** (10,300)	**37,000** (24,230)	**174,640** (123,440)
China **USD**_**2016**_ **‐million** (avoided death)	**49,400** (27,480)	**163,550** (81,650)	**19,000** (13,340)	**6,830** (4,240)	**63,380** (28,210)	**8,920** (3,960)	**21,870** (11,050)	**32,630** (13,830)	**365,580** (183,760)
Rest of Asia (ROA) **USD**_**2016**_ **‐million** (avoided death)	**30,600** (19,810)	**26,300** (9,920)	**13,150** (8,530)	**4,230** (2,700)	**38,590** (18,320)	**2,270** (710)	**9,420** (4,720)	**22,720** (10,950)	**147,280** (75,660)
Europe **USD**_**2016**_ **‐million** (avoided death)	**32,500** (8,650)	**83,650** (20,410)	**37,200** (9,530)	**4,670** (1,490)	**27,880** (7,100)	**6,950** (1,750)	**26,750** (6,150)	**33,200** (8,280)	**252,800** (63,360)
North America **USD**_**2016**_ **‐million** (avoided death)	**66,850** (10,810)	**45,600** (6,130)	**7,000** (1,510)	**1,060** (210)	**35,210** (5,020)	**5,700** (710)	**4,400** (820)	**11,830** (1,810)	**177,650** (27,020)
Central America and Caribbean **USD**_**2016**_ **‐million** (avoided death)	**4,850** (2,000)	**1,500** (560)	**1,400** (570)	**360** (150)	**6,140** (1,810)	**250** (70)	**980** (310)	**4,850** (1,360)	**20,330** (6,830)
Northern Africa and the Middle East **USD**_**2016**_ **‐million** (avoided death)	**3,700** (1,270)	**11,710** (3,340)	**5,360** (2,130)	**2,710** (1,320)	**3,420** (930)	**610** (130)	**4,100** (1,360)	**33,350** (11,790)	**64,960** (22,270)
Sub‐Saharan Africa **USD**_**2016**_ **‐million** (avoided death)	**8,150** (7,920)	**400** (400)	**400** (330)	**260** (260)	**2,940** (1,850)	**140** (100)	**550** (290)	**420** (300)	**13,260** (11,450)
Total **USD**_**2016**_ **‐million** (avoided death)	**245,600** (115,330)	**347,200** (133,620)	**110,900** (56,470)	**23,500** (13,190)	**204,400** (79,780)	**25,500** (7,820)	**83,400** (35,000)	**176,000** (72,550)	**1,216,500** (513,760)

*Note.* Health benefits are in millions USD_2016_ and are shown in bold. The total numbers of avoided mortality are presented in parentheses for each region and emission.

We estimate a health burden of 513,700 avoided mortality over the northern hemisphere, and an appraised monetized health burden of above 1.2 trillion USD_2016_ for a 10% reduction in all emissions (surface and elevated emissions, all natural and anthropogenic sources; Table 1). The largest total health burden estimates are found to be in China and India, with nearly 184,000 and 124,000 annual avoided mortality for a 10% reduction in all emissions, representing nearly 60% of the hemispheric burden of mortality. Monetized health benefits in China and India are valuated to be approximately 366 billion USD_2016_ (3.1% of the GDP_China_) and 175 billion USD_2016_ (7.7% of the GDP_India_), respectively. These amount to nearly 45% of the hemispheric total health benefits. Total health benefits in Europe exceed 252 billion USD_2016_ (1.2% of the GDP_Europe_) and are estimated to be approximately 177 billion USD_2016_ (1% of the GDP_North America_) for North America.

The largest health benefits from primary PM_2.5_ emissions for a 10% reduction are found to be in North America and China, at estimates exceeding 100 billion USD_2016_ (combined surface and elevated sources). However, the highest avoided mortality estimates for a 10% reduction in primary PM_2.5_ emissions are estimated in India and China (∼54,000 and ∼55,600). Total health benefits and avoided premature mortality for a 10% change in NH_3_ emissions are found to be the largest in China across the regions (∼172 billion USD_2016_ and 85,600 deaths). The total health burden estimates (i.e., monetized health benefits and avoided mortality) from NO_X_ and SO_2_ emissions are lower in North America, compared to other regions (Table 1). The estimates for a 10% reduction in SO_2_ emissions in North Africa and the Middle East show larger health benefits and avoided mortality compared to other pollutant emissions, particularly for elevated source emissions. The lowest health benefits and avoided mortality estimates are found to be in the Sub‐Saharan Africa region, showing the highest estimates from primary PM_2.5_ emission at the surface.

### 3.2 Sectoral Contribution

**Figure 2:**
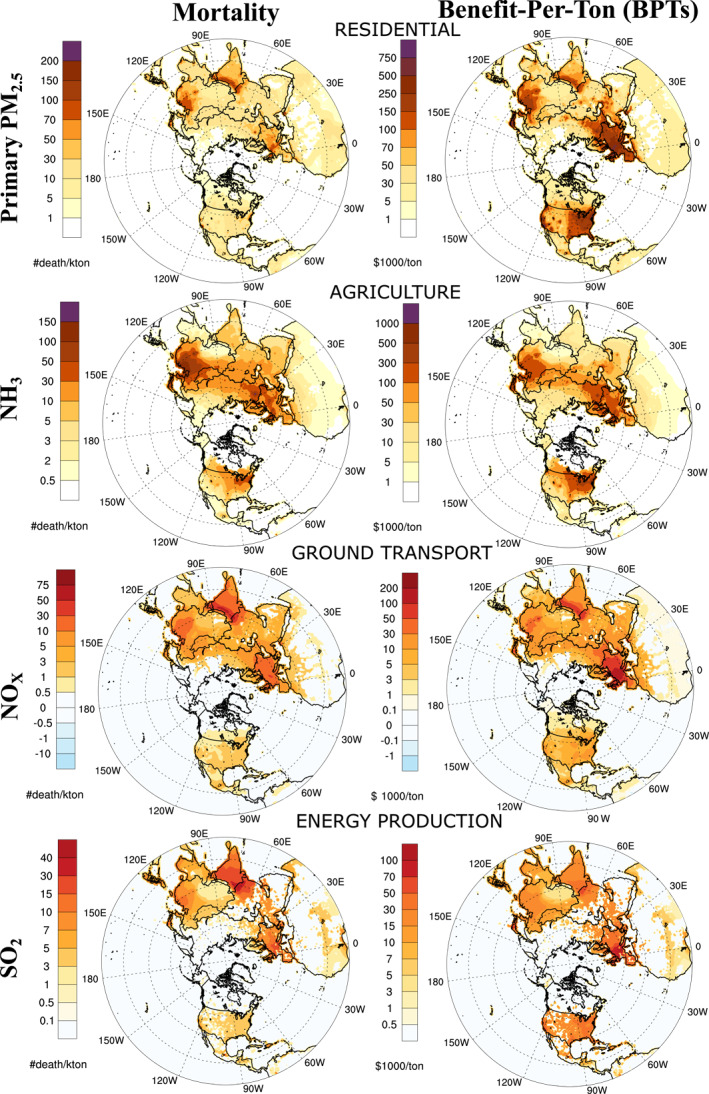
Sector‐specific annual mortality sensitivities (left‐side panel; deaths/kton) and BPTs (right‐side panel) (USD_2016_). For each emitted species, source impacts from the main contributing sectors are shown, corresponding to residential emissions, agriculture, road transportation, and energy, for primary PM_2.5_, NH_3_, NO_X_, and SO_2_ emissions, respectively.

**Figure 3:**
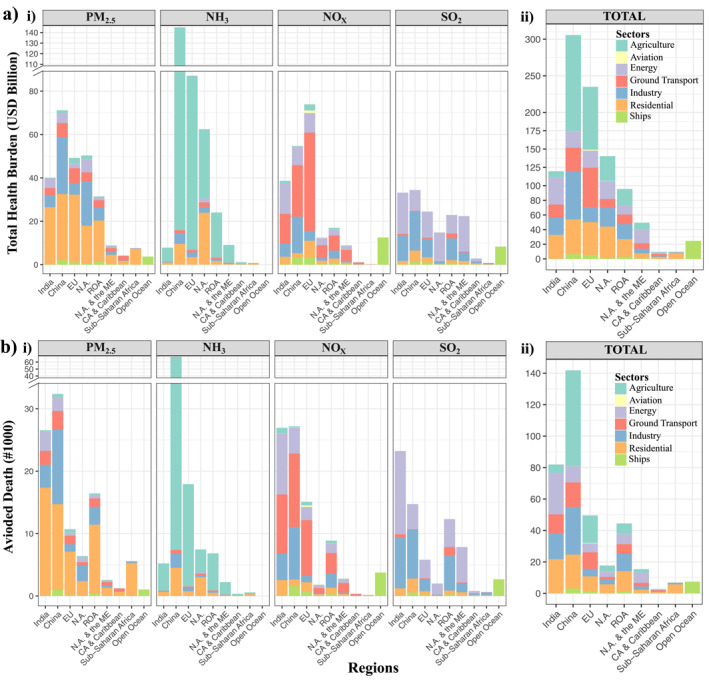
The total monetized health benefits (USD_2016_) (a) and the numbers of avoided mortality (b) for a 10% reduction in primary PM_2.5_ and precursor emissions for selected subregions in the modeling domain. Colors are indicated for each sector, indicating the sectoral contribution to total health burden for a 10% reduction in emissions. Note that energy sector includes electricity generation and energy production. Abbreviations used in the figure: E.U. for Europe; N.A., North America; ROA, Rest of Asia; N.A. & the ME, Northern Africa and the Middle East; CA & Caribbean, Central America and Caribbean.

We assess sector‐specific BPT and mortality sensitivities for seven main anthropogenic emission sectors (Task Force on Hemispheric Transport Air Pollution (TF HTAP) inventory v2 sectors). Figure 2 shows a sample of the sensitivities for the four species in their respective sectors of their dominant emissions (e.g., NO_X_ sensitivities for ground transport sector). We calculate the contribution of each anthropogenic source category to monetized health benefits and avoided mortality for each of selected subregions (Figure 3), and additionally determine the fractional contribution of the sectors to these health impacts over northern hemisphere and GBD subregions (see Figure S8 in Supporting Information S1). Sector‐specific health impacts (BPTs and mortality estimates) show the effectiveness of sectoral regulations. The emissions are attributed to seven anthropogenic source categories which correspond to ∼70% of overall avoided mortality and nearly 80% of overall total health benefits, with the balance attributable to natural and biogenic sources. We estimate that 10% emission reductions in these seven sectors results in 369,000 avoided mortality and approximately 989 billion USD_2016_ in total health benefits.

Within the seven anthropogenic sectors, the agricultural sector is the most impactful emission source category in the northern hemisphere, presenting above 95,000 deaths (19% of overall avoided mortality) and more than 290 billion USD_2016_ health benefits (24% of overall health benefits) for a 10% reduction in emissions (Figure S8 in Supporting Information S1). NH_3_ is the dominant emission contributing to health impacts from agriculture, rather than the primary PM_2.5_ emissions from the sector. Large agricultural contributions are found in China and Europe, followed by the Rest of Asia (ROA), India, and North America (Figure 3).

The second largest health burden is from residential emissions, contributing 16% of overall avoided mortality and 17% of total health benefits. Primary PM_2.5_ emissions from the residential sector contribute to a larger proportion of health impacts, compared to inorganic precursor emissions in Asia, particularly in India and China. The energy production sector accounts for 12% of overall avoided mortality and total health benefits. Similarly, the industrial sector contributes about 13% and 14% to overall avoided mortality and total health benefits, respectively. As expected, SO_2_ is the dominant contributor to health impacts from energy and industrial categories, while the contribution of NO_X_ and primary PM_2.5_ emissions from these sectors is also significant, specifically in India and China (Figure 3).

Ground transport emission reductions are responsible for 10% of overall avoided mortality and 12% of total health benefits. As expected, the largest contribution is from NO_X_ emissions, while primary PM_2.5_ emissions represent a lower, but sizeable portion of avoided mortality and health benefits. The contribution of the ground transport sector to the number of avoided mortality from NO_X_ emissions in India, China and Europe is larger than in other regions; however, among these regions, contribution from NO_X_ emissions to the total health benefits is significantly higher in Europe, followed by China and India (Figure 3). The health impact of the aviation sector is the lowest among the sectors, contributing less than 1%. The shipping sector contributes to nearly 3% of overall avoided mortality and total health benefits (Figure S8 in Supporting Information S1). Shipping emissions are evaluated in two categories: the one of the inland ship emissions from lakes, rivers, and coastal regions of countries, and the other one of the international shipping emissions over open ocean locations. Inland shipping contribution is found to be largest in China and ROA, while international shipping emissions show a proportionally larger contribution.

## 4 Discussions

In this study, we examine the long‐term PM_2.5_‐related health impacts using adjoint sensitivity analysis to attribute location‐specific BPT estimates and avoided mortality to primary PM_2.5_ and its precursor emissions (SO_2_, NO_X_ and NH_3_) over the northern hemisphere. While there are numerous previous studies on air pollution health impacts at the global or hemispheric scale (e.g., Wang et al., 2017), our adjoint approach provides more granular details in location‐specific impacts. The spatial distribution of source impact estimates shows large variability. Source impacts from primary PM_2.5_ emissions mostly follow the population distribution, unlike precursor emissions. Source impacts from inorganic precursor emissions are governed by atmospheric circulation and the formation of inorganic aerosols. Negative source impacts for NO_X_ are found in some regions, indicating that reducing emissions may cause a slight increase to the health burden at the onset of pollution control measures; however, this is limited to locations such as highly populated cities with more prevalent NO_X_‐inhibited chemical regimes. We also find significant seasonal variability in source impacts due to changes in atmospheric conditions over a year, particularly in secondary inorganic emissions, similar to the findings of Zhao et al. (2024).

Spatial distributions of the primary PM_2.5_ mortality sensitivities and BPTs generally follow that of the population density. Mortality sensitivities (deaths/kton) from primary PM_2.5_ emissions are similar in size in China and northern India and most parts of Europe while the mortality sensitivities in North America are found to be lower due to smaller populations. On the other hand, BPT estimates for primary PM_2.5_ emissions show the most sizable influences over Europe and North America, indicating high VSL regions; however, they were in a comparable range to the BPT estimates over China and northern India.

There are ethical concerns about using different VSLs for different countries. Previous studies argue that one uniform VSL should be applied to a global policy to address ethical concerns (West et al., 2006, 2012). However, several studies indicate that a larger proportion of health burdens result from emissions within the same nation with limited cross‐border transport (Anenberg et al., 2014; Liu et al., 2009; Nawaz et al., 2023; West et al., 2013). Therefore, using country‐specific VSLs is not likely to change country‐specific burden or source impact estimates in a significant way, particularly for larger countries whose spatial extent spans over a large number of grid cells. More importantly, use of country‐specific VSLs and generation of location‐specific BPTs has practical advantages for providing insights into cost‐benefit analysis of regional and global measures.

We estimate a hemispheric burden of 513,700 avoided mortality, valuated at above 1.2 trillion USD_2016_ for a 10% reduction in hemispheric emissions (all natural and anthropogenic sources). Our results can be extrapolated linearly (to 100% reduction) to compare with earlier estimations; however, such linear extrapolation may entail significant error for species whose response is likely to exhibit strong nonlinearity (e.g., NH_3_ and NOx). We note that due to the concave shape of the employed CRF, the burden estimates for subsequent 10% reductions are likely to be larger, resulting in our linear extrapolation to full burden to be an underestimation. In addition, reductions that result in concentrations below 2.4 μg/m^3^ would cause no additional mortalities in the GEMM concentration response function due to the imposed limit (*C*_*f*_ = 2.4). We find the largest proportion of mortality in East Asia and South Asia, particularly in China and India, and the lowest proportion in High‐income North America.

Our extrapolated burden estimate for the northern hemisphere (5.14 million) compares well with literature values for global burdens that range from 3.2 million to 8.53 million deaths (Apte et al., 2015; Burnett et al., 2018; Cohen et al., 2017; Lelieveld et al., 2015, 2019; McDuffie et al., 2021). Previous results support this direct comparison by showing that 80%–95% of the burden is in the northern hemisphere (Apte et al., 2015; Burnett et al., 2018; Cohen et al., 2017; Lelieveld et al., 2015, 2019; McDuffie et al., 2021).

In comparison with past studies of valuated burden, our results show somewhat higher estimates. Our estimated valuated burden in the northern hemisphere is $1.2 trillion USD, or an extrapolated $12 trillion USD for if all emissions were fully reduced. The OECD estimates the monetized health burden due to ambient PM_2.5_ concentrations as 2.85 trillion USD (2010 PPP) in 2016 and expects it to increase to 16–23 trillion USD by 2060 over the northern hemisphere (OECD, 2016). A joint report of the World Bank and IHME (The World Bank and IHME, 2016) calculates the total hemispheric valuated burden of ambient PM_2.5_ as ∼3.3 trillion USD (2011 PPP) for the year 2013. A recent report from World Bank estimates the monetized health burden due to exposure to ambient PM_2.5_ to be ∼6.13 trillion USD (PPP‐adjusted) in 2019 over the northern hemisphere (The World Bank, 2022).

Our monetized health burden estimates are larger than previously reported values. Firstly, the GEMM risk function estimates health burden is about 120% higher than the GBD risk function used in previous valuation studies (integrated exposure risk, IER) (Burnett et al., 2018). This difference is evident in the finding of McDuffie et al. (2021), where the burden estimated using GEMM was 62% larger than that estimated using GBD. Furthermore, extrapolating burden estimates from a 10% emission reduction to a 100% reduction poses additional challenges, particularly for precursor emissions. In addition to nonlinearity in atmospheric response, the adjoint estimates are for the higher range of concentrations at each location (i.e., the beginning of the emission reduction path) where the slope of the CRF is lower. Therefore, extrapolation to full mortality range is likely to result in significant underestimation. For this reason, we provide our estimates for 10% emission reductions, recognizing that full extrapolation is not appropriate. For the same reason, burden estimates from GEMM in previous studies (e.g., Burnett et al., 2018), are likely to be inaccurate if interpolated to smaller changes in emissions/concentrations. We also note that our model performance indicates that the simulated PM_2.5_ concentrations are considerably underestimated in China and India. Due to the concave form of GEMM, this can lead to an overestimation of mortality sensitivities and BPTs in these regions.

The sensitivities from the adjoint simulation for a specific location can produce the same health impact regardless of the type of emission sources; however, the health impact can be different for sectoral emissions due to their temporal and spatial variability. This also helps to create sector‐specific and pollutant‐specific emission control strategies. Therefore, we estimate sector‐specific contribution to total avoided mortality and total health benefits for a 10% reduction in sectoral emissions for GBD regions over the northern hemisphere (Figure S8 in Supporting Information S1). Note that the percentages of contributions for anthropogenic sectors are calculated using location‐specific BPT estimates and avoided mortality per ton of reduction in PM_2.5_ and secondary inorganics. Studies in the literature estimating the sectoral contribution to the health burden are based on scenario‐based sensitivity analysis. These sensitivity simulations are performed by removing emissions from individual source sectors one at a time (zero‐out method). However, removing the emissions from one sector and estimating the contribution with a scenario‐based approach can cause a large change in the concentrations, resulting in misleading source contributions in presence of a nonlinear CRF such as GEMM or IER. In that sense, the order of reduction in scenarios would affect the estimation, because of nonlinearity in atmospheric transformations. On the other hand, our results are based on marginal reduction in each emission source location, and are not sensitive to large scale changes. In comparing adjoint‐based results with scenario‐based estimates, one should take note of this important distinction. In practice, emission reductions do target specific pollutants (e.g., NO_X_) or sectors (e.g., EGU), and therefore, the actual concentration response will be sensitive to how reductions are pursued.

Comparison of sectoral impacts between different studies is limited by methodological differences in the studies such as the calculated year, grid‐spacing, use of different CTMs, CRFs, emissions, population, and mortality rates, reflecting the variability in the results. Nevertheless, sectoral fractional contributions are compared with recent studies in the literature (Table 2). At a hemispheric scale, we find the largest contribution to the total health benefits and total avoided mortality is from the agricultural sector. The fractional contribution from the residential sector is slightly lower than in previous studies, while energy, industry, and ground transport sectors are in agreement with the existing literature. Overall, the relative ranking of sectoral contributions in this study is consistent with Nawaz et al. (2023), who also employed the HTAP emissions inventory and found a similar hierarchy among major anthropogenic sectors.

**Table 2:** The Fractional Contribution of Seven Main Source Categories to Total Avoided Mortality (%)

Sector	This study (%)	Lelieveld et al. (2015)	McDuffie et al. (2021)	Weagle et al. (2018)	Silva et al. (2016)	Anenberg et al. (2019)
Agriculture	19	20	8	9	–	–
Residential	15	31	20	21	30	–
Industry	13	7	11.7	18	14.5	–
Energy	11	14	10.2	15	13	–
Ground Transport	9.5	5^a^	6.9	8^b^	9.5	10
Aviation	<1	–	–	–	<1	–
Shipping	2.5	–	0.7	–	<1	2^c^

*Note.* The contributions are not reaching 100%. Note that the study year and source categories evaluated in the studies may differ from each other. The values show common source categories for the purpose of comparison.

^a^ Only land traffic was reported.

^b^ All transport sector reported.

^c^ Only international shipping was reported.

It should be noted that our estimates for northern hemisphere exclude Volatile Organic Compounds (VOCs) related impacts and we do not provide VOC BPT and avoided mortality estimates. This is mainly because the version of CMAQ used in the adjoint model does not include the most updated oxidation pathways that contribute to the formation of secondary organic aerosols (SOA; Appel et al., 2021; Murphy et al., 2017; Pye et al., 2017) from both anthropogenic and natural sources. Furthermore, VOC composition varies widely across different sectors, and since individual VOC species can have significantly different source impacts (BPTs and avoided mortality) values, it is not possible to report a single set of location‐specific VOC BPTs that would be applicable across multiple sectors.

The results of this study are subject to certain limitations. First, our health burden estimates are for the northern hemisphere only, so the values presented are not global. The location‐specific avoided mortality and BPT estimates in the study can be influenced by uncertainties arising from air quality and meteorological models applied, exposure‐response functions, and emission inventory used. Another limitation of this study is that health burden estimates are only for PM_2.5_‐related mortality, not considering exposure to O_3_ or other pollutants. In addition, the study examines the mortality due to exposure to PM_2.5_; however, other metrics such as DALYs could also be applied. Despite the limitations acknowledged above, our study is unique in providing location‐specific source impacts across the northern hemisphere. This approach could help provide valuable information for a cost‐benefit analysis of prospective air quality regulations.

## 5 Conclusion

In this study, location‐specific BPTs and mortality sensitivities are estimated using the adjoint of CMAQ. Estimating location‐specific source impacts and resolving their spatial variability is invaluable as it provides unique information in assessing current air quality regulations and the development of future strategies for managing air quality. Furthermore, assessing sector‐specific source impacts and evaluating sectoral prevalence across various regions provides insights into understanding the impact of the different types of emission sources within specific areas, ultimately facilitating the development of effective mitigation measures. Economic valuation in international scale, despite the associated limitations and ethical concerns, can serve as a useful tool in decision‐making at both regional and global scales. This is particularly true in the context of decarbonization, by offering a direct means to cost‐benefit analysis or trade‐offs associated with various regulations and strategies. Monetized health outcomes can be useful within the policy and regulatory context, as they provide quantifiable insights regarding the economic implications.

## Disclaimer

The views expressed in this paper are those of the authors and do not necessarily represent the view or policies of the U.S. Environmental Protection Agency.

## Conflict of Interest

The authors declare no conflicts of interest relevant to this study.

## Supporting information

Supporting Information S1

## Data Availability

Software—The source code of adjoint of Hemispheric CMAQ is available at Oztaner et al. (2025). Data—Baseline mortality rates are available at IHME (2021). Data — Gridded population data is available for download at WorldPop (2021). Data—Country‐specific GDP per capita, the consumer price index and the income growth rate are available at The World Bank (2021). Table S1 and Figure S9 in Supporting Information S1, PM_2.5_ monitor data are available for NAPS database (Canada) at ECCC (2019), for U.S. EPA AQS at US EPA (2021), for China's Air Quality Stations at Berman (2017), and India's National Air Quality Stations at CPCB (2022).
