# Supplementary material for: Source Attribution of PM2.5 Health Benefits Over Northern Hemisphere Using Adjoint of Hemispheric CMAQ
Source: Geohealth. 2026 Jan 20;10(1):e2025GH001533. doi: 10.1029/2025GH001533 (PMC12820424; doi:10.1029/2025GH001533)
Supplement: Supplementary file 1 — Supporting Information S1 [file GH2-10-e2025GH001533-s001.docx]

*GeoHealth*

Supporting Information for

**Source Attribution of PM_2.5_ Health Benefits over Northern Hemisphere Using Adjoint of Hemispheric CMAQ**

Y.B. Oztaner^1^, S. Zhao^1^, B. Henderson^2^, R. Mathur^2^, and A. Hakami^1^*

^1^Department of Civil and Environmental Engineering, Carleton University, Ottawa, ON K1S 5B6, Canada

^2^U.S. Environmental Protection Agency, Research Triangle Park, NC, USA

**Introduction**

This file contains 9 supplementary figures and 1 table. Further information is provided on adjoint model evaluation with finite difference method (FDM) (Figures S1 and S2), estimated health impacts (BPT and mortality sensitivities) of elevated emission sources for primary PM_2.5_ and precursor PM_2.5_ emissions (Figure S3), estimated seasonal mortality (Figures S4-5) and BPT (Figures S6-S7) sensitivities for primary PM_2.5_ and precursor PM_2.5_ emissions, and fractional contributions of source categories for total health benefits and total avoided deaths for 10% reduction in primary PM_2.5_ and inorganic precursor emissions in 2016 (Figure S8). Model performance evaluation, along with regional daily average observed and modeled concentrations, is presented in Table SI-1 and Figure S9.

**Figure S1.** ADJ (adjoint) vs FDM sensitivities of the final NTR (7 species) concentrations (ppmV) with respect to all the initial NTR concentrations (ppmV) from a 1-day test simulation. The perturbation size is (a) 0.1 ppb for FDM (R^2^: 0.978), (b) 0.01 ppb. (R^2^: 0.993), and (c) 0.001 ppb (R^2^: 0.989).

**Figure S2.** ADJ (adjoint) vs FDM sensitivities of the final O_3_ concentrations (ppmV) with respect to the initial NO_2_ concentrations (ppmV) from a 1-day test simulation. The perturbation size is (a) 0.1 ppb for FDM (R^2^: 0.949), (b) 0.01 ppb (R2: 0.992) and (c) 0.001 ppb (R^2^: 0.993).

**Figure S3.** Estimated annual BPTs (USD_2016_) for a reduction in primary PM_2.5_ and PM_2.5_ precursor emissions (NH_3_, NO_X_ and SO_2_) emitted from elevated sources (left panel) and estimated annual mortality counts (number of deaths) attributable to a kton reduction in primary PM_2.5_ and its precursor emissions in each location (right panel).

**Figure S4.** Estimated seasonal mortality sensitivities (deaths/kton) for a reduction in primary PM_2.5_ and NH_3_ emissions from surface sources across the Northern Hemisphere.

**Figure S5.** Estimated seasonal mortality sensitivities (deaths/kton) for a reduction in NO_X_ and SO_2_ emissions from surface sources across the Northern Hemisphere.

**Figure S6.** Estimated seasonal BPTs (USD_2016_) for a reduction in primary PM_2.5_ and NH_3_ emissions from surface sources across the Northern Hemisphere.

**Figure S7.** Estimated seasonal BPTs (USD_2016_) for a reduction in NO_X_ and SO_2_ emissions from surface sources across the Northern Hemisphere.

Figure S8: Fractional contributions of source categories to a) attributable monetized health benefits, and b) total avoided deaths for 10% reduction in primary PM_2.5_ and inorganic precursor emissions in 2016. The values at the left of each bar demonstrate the total health burden for health benefits and avoided premature deaths. Note that energy sector includes electricity generation and energy production.

**Model Performance**

Model performance metrics such as mean bias (MB), normalized mean bias (NMB) mean absolute error (MAE) are applied to simulated and observed PM2.5 concentrations. Modeled PM2.5 concentrations are aggregated to match with observations and metrics are calculated for model-observation pairs in time and space.

The daily average PM2.5 observations for simulation period were retrieved from the NAPS database (for Canada) and the US EPA’s Air Quality Stations (AQSs) database, European Union’s AirBase database, China’s and India’s National Air Quality Stations database to conduct model performance analysis. The model performance analysis was applied for all stations which have 70% or more data availability. Simulated and observed values indicates high performance when the metrics get closer to 0.

Table SI-1 shows the model performance results. The number of model-observation pairs varies in each database. The simulated PM_2.5_ tends to be underestimated when considering all databases (domain-wide), considerably in China and India. Figure SI-9 presents the daily average modeled and observed PM_2.5_ concentrations by each location. The simulated PM_2.5_ over India indicates strong bias all over the year.

**Table SI-1**: H-CMAQ Model Performance

| Locations | N # | MB (ug/m3) | NMB (%) | MAE (ug/m3) |
| --- | --- | --- | --- | --- |
| CHINA | 117741 | -18 | -41 | 22 |
| CONUS  (NAPS+EPA’s AQS) | 96450 | 0.5 | 7 | 3.3 |
| EUROPE | 124458 | -5.1 | -39 | 6.2 |
| INDIA | 2946 | -47 | -55 | 49 |
| ALL | 341595 | -8.3 | -37 | 11 |


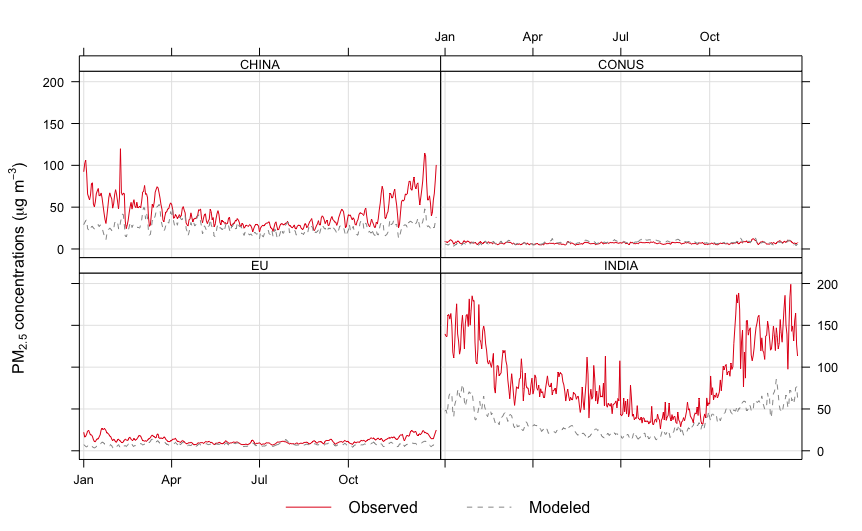


Figure S9: Time series of daily average observed and modeled PM_2.5_ for regions
